# Dengue virus specific IgY provides protection following lethal dengue virus challenge and is neutralizing in the absence of inducing antibody dependent enhancement

**DOI:** 10.1371/journal.pntd.0005721

**Published:** 2017-07-07

**Authors:** Ashley L. Fink, Katherine L. Williams, Eva Harris, Travis D. Alvine, Thomas Henderson, James Schiltz, Matthew L. Nilles, David S. Bradley

**Affiliations:** 1 Department of Biomedical Sciences, School of Medicine and Health Sciences, University of North Dakota, Grand Forks, North Dakota, United States of America; 2 W. Harry Feinstone Department of Molecular Microbiology and Immunology, The Johns Hopkins Bloomberg School of Public Health, Baltimore, Maryland, United States of America; 3 Division of Infectious Disease and Vaccinology, School of Public Health, University of California, Berkeley, Berkeley, California, United States of America; 4 Avianax, LLC, Grand Forks, North Dakota, United States of America; University of Texas Medical Branch, UNITED STATES

## Abstract

Dengue hemorrhagic fever (DHF) and dengue shock syndrome (DSS) are severe disease manifestations that can occur following sequential infection with different dengue virus serotypes (DENV1-4). At present, there are no licensed therapies to treat DENV-induced disease. DHF and DSS are thought to be mediated by serotype cross-reactive antibodies that facilitate antibody-dependent enhancement (ADE) by binding to viral antigens and then Fcγ receptors (FcγR) on target myeloid cells. Using genetically engineered DENV-specific antibodies, it has been shown that the interaction between the Fc portion of serotype cross-reactive antibodies and FcγR is required to induce ADE. Additionally, it was demonstrated that these antibodies were as neutralizing as their non-modified variants, were incapable of inducing ADE, and were therapeutic following a lethal, antibody-enhanced infection. Therefore, we hypothesized that avian IgY, which do not interact with mammalian FcγR, would provide a novel therapy for DENV-induced disease. We demonstrate here that goose-derived anti-DENV2 IgY neutralized DENV2 and did not induce ADE *in vitro*. Anti-DENV2 IgY was also protective *in vivo* when administered 24 hours following a lethal DENV2 infection. We were also able to demonstrate via epitope mapping that both full-length and alternatively spliced anti-DENV2 IgY recognized different epitopes, including epitopes that have not been previously identified. These observations provide evidence for the potential therapeutic applications of goose-derived anti-DENV2 IgY.

## Author summary

Dengue hemorrhagic fever (DHF) and dengue shock syndrome (DSS) are severe disease manifestations following secondary heterotypic dengue virus (DENV) infections. DENV infects almost 400 million people annually and there are currently no licensed therapies to treat DENV-induced disease. DHF and DSS are mediated by serotype cross-reactive antibodies that facilitate antibody dependent enhancement (ADE) by binding to viral antigens and then Fcγ receptors (FcγR) on surrounding cells. ADE results in a heightened immune response and in part mediates the pathogenesis of secondary DENV infections. Researchers have developed an animal model of ADE-induced severe DENV in which anti-DENV2 antibodies genetically engineered to eliminate FcR binding have therapeutic and prophylactic efficacy. Our study suggests that avian-derived anti-DENV2 IgY, without genetic modification, is able to provide protection both *in vitro* and against a lethal DENV challenge *in vivo*. IgY has emerged as an attractive, alternative therapeutic antibody because of its biological characteristics that enable it to prevent some of the adverse reactions that mammalian antibodies have when used for human immunotherapy. Specifically, IgY does not bind to mammalian complement or rheumatoid factor and has proven to be very safe for human consumption. Without modification, anti-DENV2 IgY can be rapidly produced and may serve as a good therapeutic option to treat dengue worldwide.

## Introduction

Almost half of the world is at risk for dengue virus (DENV) infections with up to 390 million infections occurring in nearly 100 endemic countries annually [1]. Dengue is a rapidly emerging disease with a 30-fold increase in disease incidence reported in the past 50 years [2]. Dengue has established itself globally in both endemic and epidemic transmission cycles and is currently regarded as one of the most important arboviral diseases internationally [1, 3, 4].

DENV is a member of the Flavivirus family of RNA viruses. There are four distinct serotypes (DENV1, DENV2, DENV3, DENV4) that differ at the amino acid level by 25–40% [5]. DENV is primarily transmitted by the *Aedes aegyptii* mosquito, and *Aedes albopictus* is a secondary vector. In the Americas, epidemic dengue was controlled in most of the region by the eradication program that eliminated the *Aedes aegypti* mosquito vector from 23 countries until the program was terminated in the early 1970s [6]. Following the termination of this program, the mosquito rapidly reestablished itself and all four DENV serotypes re-emerged, resulting in the co-circulation of multiple DENV serotypes [4]. It has become increasingly evident that in order to control the disease in the absence of a strong vector control program, the development of new antiviral therapies and vaccines is crucial.

DENV can affect people of all ages including infants, children, adults and the elderly, but the interplay between the virus and host is what determines the clinical outcome. Disease manifestations from DENV infections range from asymptomatic infections, a mild febrile illness known as dengue fever (DF), or the more severe dengue hemorrhagic fever (DHF) and dengue shock syndrome (DSS). During an initial infection, most children experience subclinical infection or mild undifferentiated febrile syndromes[7]. In this situation, lifelong immunity against the primary infecting serotype occurs. During a secondary infection, the pathophysiology of the disease can change dramatically, specifically if the secondary infection is with a different DENV serotype. Heterotypic secondary infections are the cause of 90% of the DHF/DSS cases reported [8]. One working hypothesis explaining the severity of dengue pathogenesis observed during secondary infection is antibody dependent enhancement (ADE) [7]. ADE occurs when sub-neutralizing antibodies following a primary DENV infection bind to an infecting viral particle from the secondary heterotypic infection. These antibody-virus complexes then bind to Fcγ receptors (FcγR) on macrophages and dendritic cells via the Fc portion of the antibody [9]. The result of ADE is a higher number of infected immune cells, leading to heightened immune response to the infection [9]. ADE also results when infants are born to dengue immune mothers after maternal anti-DENV antibodies have been catabolized to sub-neutralizing levels[10].

At present, there remains an unmet need for an effective dengue therapeutic that is able to shorten the duration of the illness, prevent the development of severe disease, and reduce the severity of common symptoms [11]. There are a number of institutions, both academic and pharmaceutical, that are currently engaged in the discovery and development of therapeutics [11]. One encouraging area of research has been the development of therapeutic anti-DENV monoclonal antibodies that block viral infection. Balsitis *et al*. verified using aglycosylated and F(ab’)_2_ DENV-specific IgG that the interaction between the Fc portion of the serotype cross-reactive antibodies and the FcγR is required to induce ADE. In this study, they demonstrated that the genetically modified DENV-specific antibodies did not enhance DENV2 infection and were able to neutralize DENV infection as well as the non-modified variants. Furthermore they demonstrated that these antibodies were therapeutic up to 48 hours following a lethal, antibody-enhanced infection [12]. We suggest that IgY, the avian homolog to mammalian IgG, which naturally fails to bind FcγR, could be used as alternative therapeutic antibodies to treat DENV-induced disease.

IgY is the primary immunoglobulin isotype in oviparous animals and is the functional equivalent to mammalian IgG, but also has the ability to sensitize tissues to anaphylactic reactions [13]. There are two IgY isoforms present in anseriform birds: full-length IgY and alternatively spliced IgY, which lack two of the constant regions present in full-length IgY. The alternatively spliced IgY coexists with the full-length IgY and is a structural equivalent to a mammalian F(ab’)_2_ fragment [14]. Previous studies from our laboratory have confirmed the potential therapeutic efficacy of goose-derived IgY antibodies in neutralizing viral infections and preventing Hantavirus pulmonary syndrome (HPS). Geese were vaccinated with a DNA vaccine encoding virus envelope glycoproteins, and Andes virus (ANDV)-specific IgY was isolated and purified from goose egg yolks. It was demonstrated that ANDV-specific IgY provided protection from HPS when administered to hamsters 5 days post-infection with ANDV (25 LD_50_) [15].

There are many advantages to using IgY for the treatment of DENV infections. One important advantage is the genetic background and phylogenetic distance that distinguishes birds from mammals. Avian derived IgY, as compared to mammalian IgG has a higher avidity for some mammalian antigens and has the ability to recognize different epitopes that may be non-immunogenic in mammals [16, 17]. Another key advantage of IgY is its inability to bind mammalian FcγR; therefore, it is possible that anti-DENV IgY will be able to neutralize a viral infection without inducing ADE [18]. Furthermore, IgY does not interact with other Fc-binding receptors, which limits its ability to elicit an inflammatory reaction in humans [19–24]. Although IgY is able to activate complement in the avian system, its unique structure prevents it from being able to activate human complement [23]. Although the complement component C1q has been demonstrated to increase neutralizing potential of anti-West Nile virus antibodies in the absence of inducing ADE, it has also been proposed that complement activation during secondary heterotypic DENV infection by non-neutralizing antibodies contributes to immune enhancement. Furthermore, it has been demonstrated that products of complement activation, specifically C3a, C5a, and SC5b-9 are elevated in patients with DHF [25–28]. Therefore, the inability of anti-DENV IgY to induce complement activation could prove to be detrimental or beneficial depending on the individual disease state. Furthermore, the ability of IgY to be easily isolated from the egg yolk presents another key advantage to using IgY as an alternative to mammalian IgG. Eggs can be collected from laying hens and the egg yolks used as a source of IgY. This is a rapid process that avoids serum collection while still maintaining high antibody yields. The concentration of IgY present in the egg yolk of immunized birds depends on the species, age, and antigen injected. Whereas concentrations of antibody from chicken egg yolk ranges from 60-150mg per egg, the concentrations of antibody from goose egg yolk ranges from 100-500mg per egg. Furthermore, there are a number of industrial processes setup for the collection and separation of eggs, making large-scale production of IgY a feasible option [16].

In this study, we demonstrate the efficacy of goose-derived, purified anti-DENV2 IgY *in vitro* and *in vivo*. Specifically, we demonstrate that anti-DENV2 IgY is able to neutralize DENV2 *in vitro* and provide protection in the AG129 mouse model when administered therapeutically following lethal challenge. Anti-DENV2 IgY was not able to enhance DENV2 infection *in vitro*, thereby providing protection in the absence of ADE. Finally, we confirmed that anti-DENV2 IgY binds to previously uncharacterized epitopes, and furthermore full-length IgY and alternatively spliced IgY also bind epitopes that are unique from one another.

## Materials and methods

### Ethics statement

Research was conducted in compliance with the Animal Welfare Act and adheres to the recommendations in the Guide for the Care and Use of Laboratory Animals of the National Institutes of Health. The protocol was approved by the Institutional Animal Care and Use Committee at the University of California Berkeley (R252-1012B).

### Geese

The geese (*Anser domesticus*, 25 months old) used in this study were an inbred (over 35 generations) hybrid between the German Embden, the Royal Chinese, and the Royal English breeds. An existing 10,150 sq. ft. barn at Schiltz Goose Farm, Inc. was converted into a Specific Pathogen Free (SPF) facility. 3,900 sq. ft. of the barn was reserved as a clean room for the ISO egg production units. The ISO facility had additional HEPA filtration to remove particles as small as 0.3 microns with a 99.97% minimum particle collective efficiency. Only authorized personnel were involved in the cleaning of the barn in the SPF and ISO facilities and all animal handling.

### Vaccination

Dengue Type 2 Antigen was purchased from Microbix Biosystems Inc. and used to vaccinate ten female geese. According to the manufacturer, the Dengue Type 2 Antigen was prepared by infecting Vero cells with DENV 16681 and collecting virus-containing supernatants. Virus particles were inactivated by formaldehyde at room temperature and neutralized by the addition of sodium bisulfite. Virus particles were purified by ultracentrifugation over a sucrose cushion and resuspended in Medium 199. Geese were vaccinated with 120 μg of Dengue Type 2 Antigen on day 0 and boosted with 60 μg at weeks 2 and 4. Immunizations consisted of 2 x 200 μL subcutaneous injections at the back of the neck in two different injection spots. The eggs were collected starting from week 2 after the first immunization and stored at 4°C until further use.

### Purification of IgY from goose egg yolk

Yolks were isolated and rinsed with water and then punctured to drain the contents, which were diluted 1:10 with cold deionized water, stirred, and acidified to pH 5.0. The diluted yolk was centrifuged at 10,000 x g for 30 minutes, and the supernatant was filtered. In order to separate the full-length IgY from the alternatively spliced IgY, a sequential series of 30%, 40% and 50% ammonium sulfate was used. The pellets were suspended in 50 mM Tris HCl pH 8.0. Further purification was achieved via hydrophobic charge induction chromatography on 4-Mercapto-Ethyl-Pyridine-linked (MEP) HyperCel sorbent (Pall Corporation) followed by concentration using a Tangential flow filtration and diafiltration with 1x PBS buffer.

### SDS-PAGE

Rabbit- anti-goose IgY-IgG (obtained from rabbits immunized with purified naïve goose IgY), full-length anti-DENV2 IgY, and alternatively spliced anti-DENV2 IgY were separated by 4–15% gradient SDS-PAGE under non-reducing conditions. The gel was incubated in Bio-Safe Coomassie G-250 stain (Bio-Rad Laboratories) for 30 minutes and subsequently de-stained in deionized water for 2 h. Signals were captured using the AlphaView software and AlphaImager HP Imaging System (Alpha Innotech).

### Antibody detection

The antibody activity of anti-DENV2 IgY was determined by ELISA. Briefly, microtiter plates were coated with 100 μL of the capture antigen (Dengue Type 2 Antigen, Microbix Biosystems Inc) and stored at 4°C overnight. After washing the plates 3 times with wash buffer (1X PBS, 0.05% Tween-20), they were blocked with 400 μL per well of blocking buffer (0.25% BSA, 0.05% Tween-20, 1X PBS) and incubated for 30 minutes at room temperature. The wells were washed 3 times with wash buffer and incubated with 100 μL of anti-DENV2 goose antibody (isolated from DENV2 vaccinated geese) serially diluted 1:2 down the plate in blocking buffer and incubated at 37°C for 30 minutes. Dengue virus E protein and naïve IgY control antibodies (isolated and purified from geese vaccinated with PBS) were included as standards on each plate. The plates were washed 3 times with wash buffer and blocked for 10 minutes at room temperature. Next, 100 μL of biotinylated rabbit anti-goose IgY antibody (500ng/mL, Covance Inc.) was added to each well and incubated at 37°C for 30 minutes. After washing the plates 3 times with wash buffer, the wells were blocked for 10 minutes at room temperature. Following this, 100 μL of diluted streptavidin-HRP antibody in blocking buffer (1:2,000) was added to each well, and the plates were incubated at 37°C for 30 minutes. The plates were finally washed 3 times before adding 100 μL of prepared o-phenylenediamine dihydrochloride (OPD, Invitrogen) color substrate to each well and allowed to develop for 15 minutes at room temperature. The reaction was terminated by adding 50 μL of 1N H_2_SO_4_, and the absorbance was read in a BioTek plate reader at A_490._ Data are represented as endpoint titers.

### Viruses and cell lines

DENV was propagated in C6/36 cells (ATCC), and the DENV2 D2S10 strain (passaged 4 times in C6/36 cells) was derived in the laboratory of Dr. Eva Harris at the University of California, Berkeley from the parental DENV2 PL046 Taiwanese isolate as described [29]. U937-DC-SIGN cells were obtained from A. de Silva (University of North Carolina, Chapel Hill) and grown in RPMI media (Invitrogen) at 37°C and 5% CO_2_. K562 cells (obtained from ATCC) were grown in RPMI media (Invitrogen) at 37°C and 5% CO_2_.

### *In vitro* viral neutralization and ADE studies

The neutralization and enhancement titers for anti-DENV2 and control purified polyvalent IgY sera against DENV2 D2S10 were determined. Both neutralization and enhancement experiments were performed twice, each time in duplicate. In brief, the IgY sera were diluted to a starting concentration of 2.0 mg/mL. Twelve 4-fold dilutions were mixed with equivalent volumes of DENV2 D2S10 for 45 minutes before infecting U937 DC-SIGN cells, a DENV-permissive human monocytic cell line [12]. The cells were washed two hours following infection, and then fixed and stained for DENV E protein with monoclonal antibody 4G2-Alexa 488 24 hours later. The data were analyzed by flow cytometry [12], and the dilution yielding 50% neutralization (NT_50_) was calculated using GraphPad PRISM [30, 31].

To test for potential enhancement, the serum was diluted and mixed with DENV2 as described above and used to infect K562 cells, an erythroleukemic cell line that is not naturally permissive for DENV infection, but can be infected via surface FcγRIIA when DENV virions are coated with sub-neutralizing concentrations of anti-DENV antibody. The cells were fixed, stained, and analyzed as described above 48 hours following infection [30, 31].

### *In vivo* anti-DENV2 IgY protection

The therapeutic potential of goose-derived anti-DENV2 IgY was tested using conditions that cause 100% mortality in AG129 mice. Six-to-eight week old IFN-αβR^-/-^ and IFN-γR^-/-^ (AG129) mice were administered a lethal dose of DENV2 strain D2S10 (1.0x10^7^ plaque forming units (PFU)) intravenously (i.v.) [12]. Twenty-four hours after infection, mice were injected intraperitoneally with the indicated amounts of polyclonal anti-DENV2 IgY or the positive control mouse monoclonal antibody (MAb) E60 N297Q (E60 MAb originally obtained from M. Diamond, and genetically modified using QuikChange mutagenesis (Stratagene) to abolish FcγR and C1q binding [12]) in a volume of 200 μL, or 200 μL of PBS as a negative control. Mice were followed for 10 days and observed for morbidity and mortality twice daily. Anti-DENV2 IgY was administered as 50μg, 500μg, 1mg or 2mg per injection, control naïve IgY was administered as 1mg or 2mg per injection, and the previously identified protective anti-DENV monoclonal antibody IgG E60 N297Q was administered as 20μg per injection for a positive control [12].

### Epitope mapping

Anti-DENV2 IgY epitopes were mapped using E, prM, NS1 and NS3 proteins via peptide arrays. Specifically, 11 amino acid overlapping 15mer peptides were covalently attached to a microarray slide (JPT Innovative Peptide Solutions, Berlin, Germany). All Pepstar microarray protocols were provided by JPT. Briefly, a slide sandwich containing the microarray and a dummy slide was made, separated by spacers, in order to increase the incubation environment. The primary antibody serum was incubated at 30μg/mL on the slide at 4°C overnight in a moist environment. The slide was rinsed 5 times for 4 minutes each with T-TBS, then 5 times for 4 minutes each with ultra-pure water. The slide was incubated in the fluorescently labeled (Cy5) goat-anti chicken IgY secondary antibody solution (1μg/mL, Abcam) for 45 minutes, washed 5 times with T-TBS, then 5 times with ultra-pure water, and dried using a dust-free, oil-free, high-velocity canned air. Fluorescence was measured at a pixel size of 10μm using the Genepix 4000 microarray reader. The signal intensity mean values were calculated for each sub-array and background corrected values were used for interpretation in Microsoft excel. The microarray experiment was repeated with each antibody type on three separate but identical slides; anti-DENV full length IgY, anti-DENV alternatively spliced, and control naïve IgY.

## Results

### Antibody characterization

**Fig 1:**
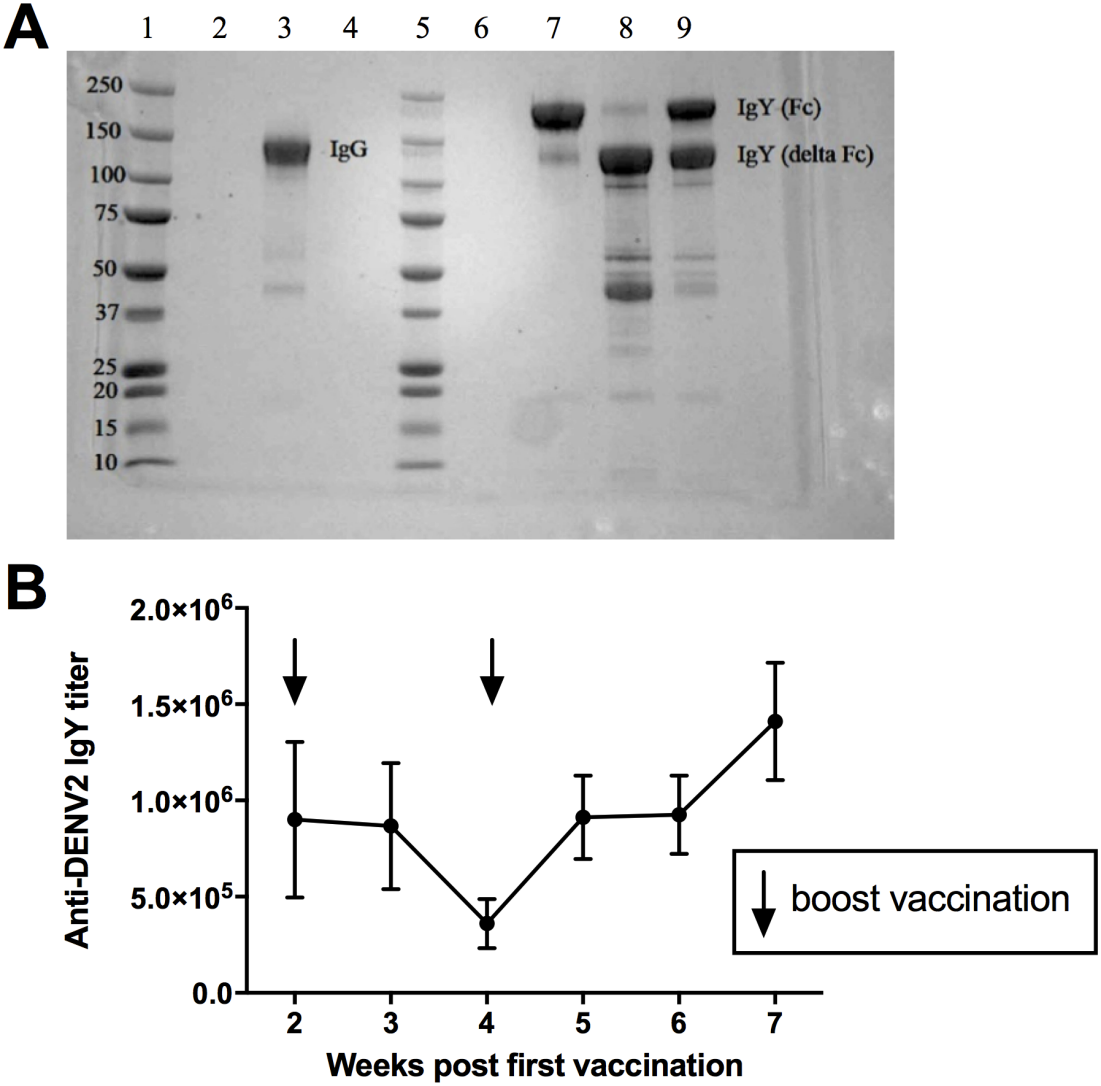
Anti-DENV2 IgY antibody characterization. **A)** Comparison of rabbit-derived IgG and goose-derived IgY isolated using mercapto-ethyl pyridine (MEP) HyperCel Hydrophobic Charged Induced Chromatography (HCIC) sorbent. Rabbit IgG was isolated from rabbits that were immunized with goose IgY. Anti-DENV2 goose IgY was isolated from egg yolks of geese immunized with DENV2 killed antigen. Separation of full length IgY and alternatively spliced IgY occurred prior to HCIC using differential ammonium sulfate precipitation (see methods). Lane 1, molecular weight marker, Lane 3, rabbit IgG anti IgY; lane 7. IgY full length; lane 8, IgY alternatively spliced and lane 9, IgY full length and alternatively spliced (2 bands). **B)** Egg yolks were collected from geese immunized with killed DENV2 virus. Arrows indicate boost immunizations at 2 and 4 weeks post-first immunization. Anti-dengue IgY antibody titer did not differ among weeks (ANOVA on ranks, p = 0.157). Data are presented as mean ± SE. N = 4, 6, 22, 24, 21, and 27 at 2, 3, 4, 5, 6, and 7 weeks post vaccination respectively.

Following the purification of anti-DENV2 IgY, a SDS-PAGE was performed to verify the purity of anti-DENV2 full length IgY and anti-DENV2 alternatively spliced IgY (Fig 1A). We were able to detect both anti-DENV2 full length (Fig 1A, Lane 7) and alternately spliced IgY (Fig 1A, Lane 8) purified antibody populations and detected both full length and alternatively spliced IgY (Fig 1A, Lane 9) in the serum that was not used to isolate the two separate antibody populations. Rabbit sera containing anti-IgY IgG was used for comparison (Fig 1A, Lane 3). An ELISA was then performed to confirm the presence of DENV2-specific IgY and to determine the antibody titer in each egg yolk. Goose egg yolk titers are considered indicative of serum titers because antibodies are transferred from the serum of the laying hen to the egg yolk during embryogenesis. Of the 104 eggs that were measured, the average serum endpoint titer across all weeks post vaccination was 1:924,807 with the highest titer being 1:6,400,000 at 7 weeks post vaccination (Fig 1B).

### *In vitro* virus neutralization and ADE

**Fig 2:**
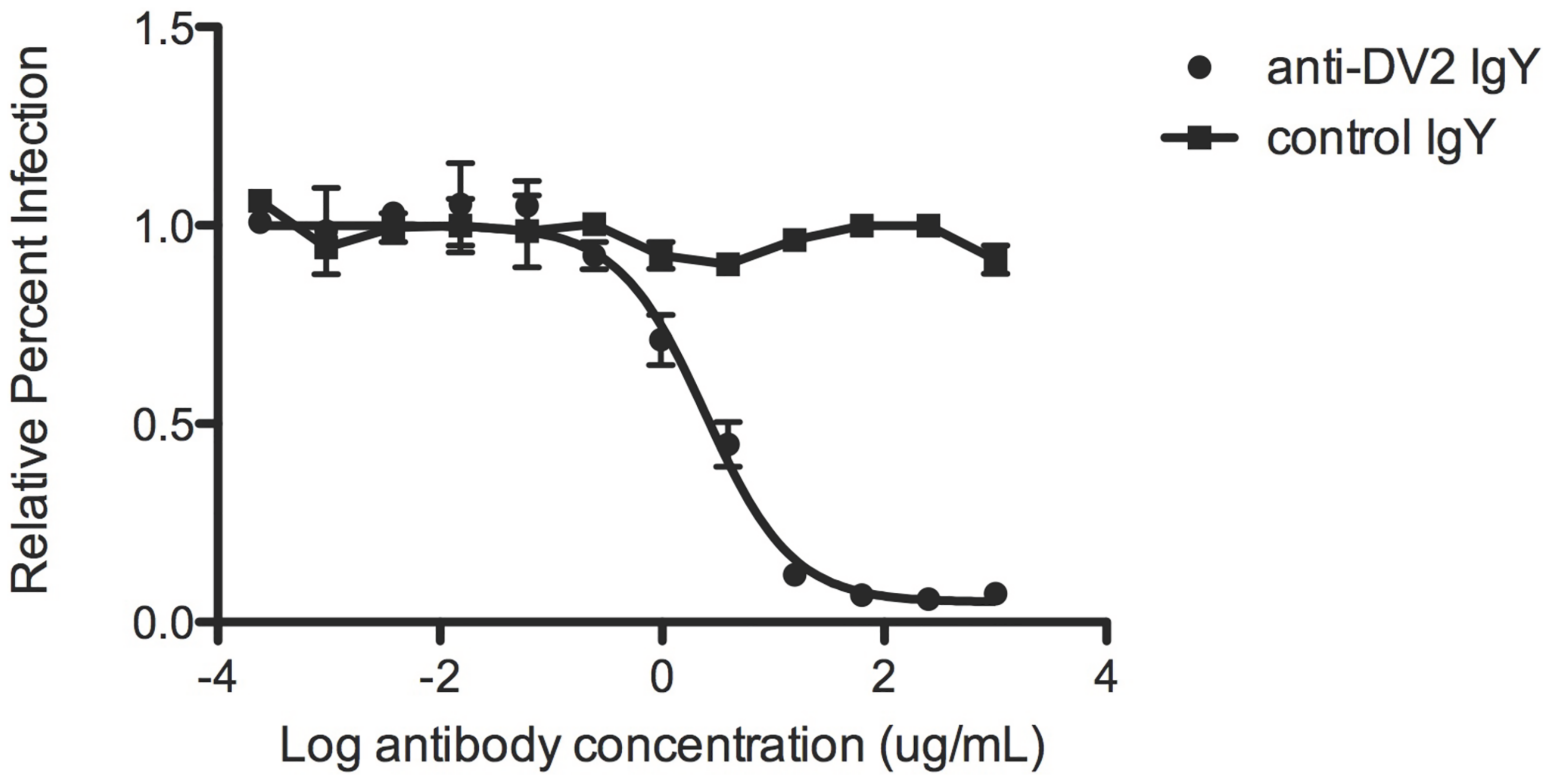
Anti-DENV2 IgY neutralizes DENV2 D2S10 *in vitro*. Purified, anti-DENV2 IgY (NT_50_ 2.6μg/mL), but not control purified IgY, neutralized DENV2 D2S10. Relative percent infection is shown on the y-axis, and log reciprocal antibody concentration on the x-axis. The data are representative of two independent experiments.

**Fig 3:**
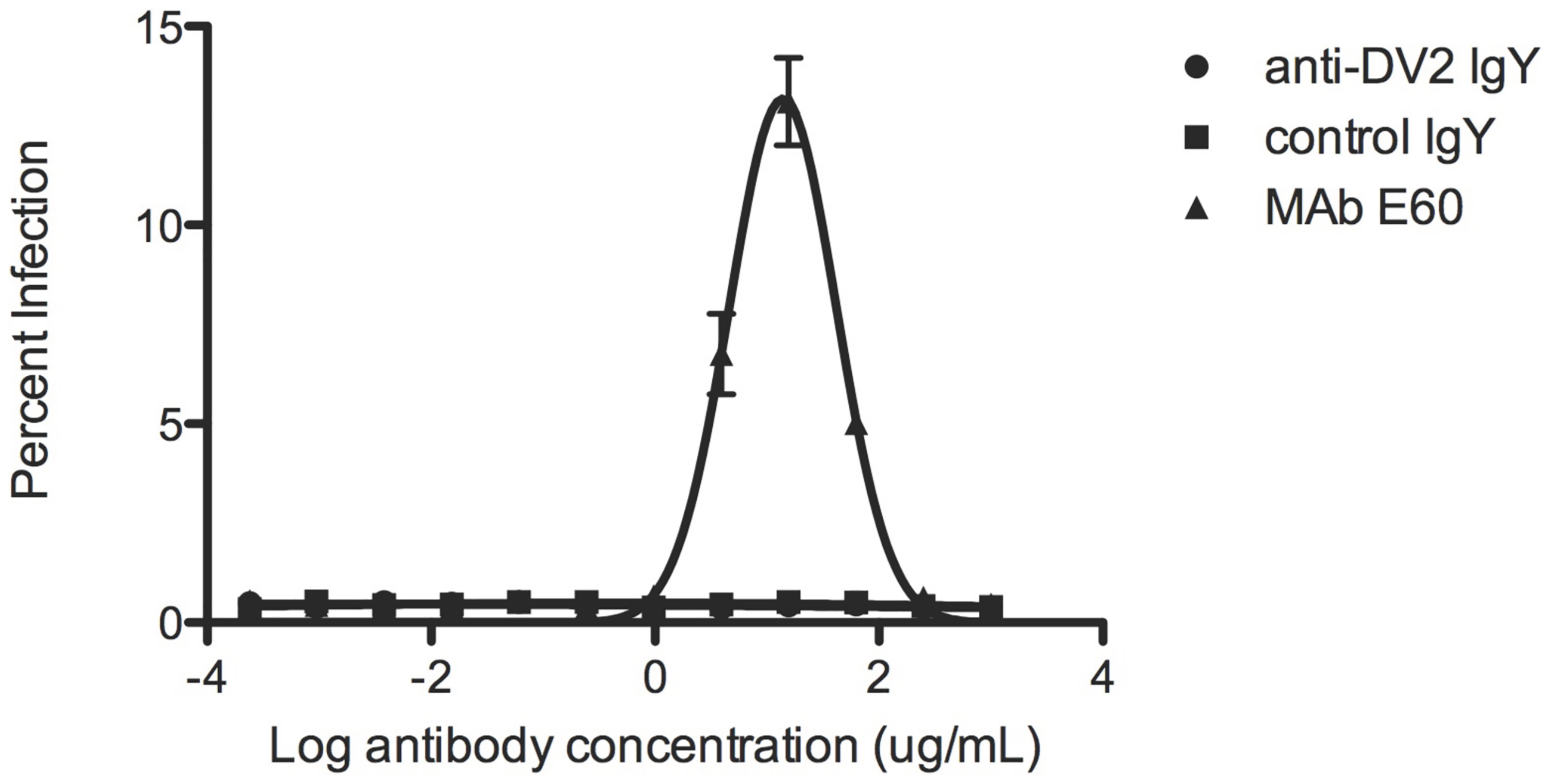
Anti-DENV2 does not enhance D2S10 infection *in vitro*. Neither purified anti-DENV2 IgY nor control purified IgY enhanced DENV2 D2S10, whereas control MAb E60 generated ~15% enhancement at the peak enhancing titer. Percent infection is shown on the y-axis and log reciprocal antibody concentration on the x-axis. These data are representative of two independent experiments.

Polyvalent anti-DENV2 IgY were purified from serum of DENV2-immunized geese. These antibodies were tested for potential neutralization and enhancement *in vitro*. Serial dilutions of anti-DENV2 IgY were mixed with DENV2 D2S10 and used to infect DENV-permissive monocytic U937 DC-SIGN cells (neutralization) or an erythroleukemic cell line, K562 (enhancement), which are not naturally permissive for DENV infection, but can be infected via surface FcγRIIA when DENV virions are coated with sub-neutralizing concentrations of anti-DENV antibody. The dilution of anti-DENV2 IgY serum yielding 50% neutralization (NT_50_) was between 1.0 and 2.6 μg/mL in two independent experiments (Fig 2). The control IgY serum did not yield a measurable NT_50_ titer in either experiment. Previous studies have identified the NT_50_ titer of anti-DENV MAb E60 and its modified variant E60 N297Q as 49 ng/mL and 72 ng/mL, respectively [31, 32]. In the enhancement experiment, whereas the positive control anti-DENV Mab E60 generated ~15% infection at its peak enhancement titer, neither the anti-DENV IgY nor control IgY sera were enhancing at any dilution tested (Fig 3).

### *In vivo* anti-DENV2 IgY protection

**Fig 4:**
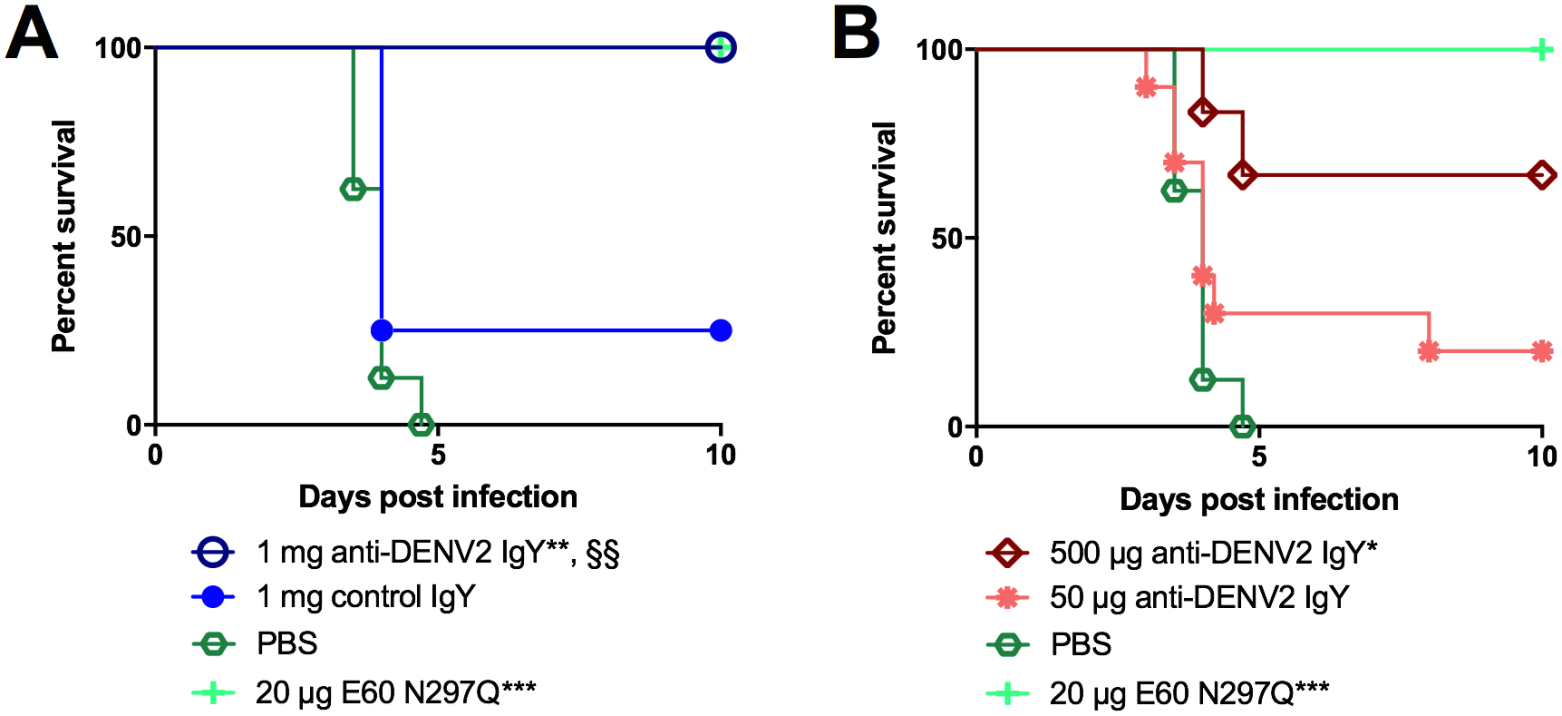
Therapeutic efficacy of anti-DENV2 IgY *in vivo*. A, B) Six-to-eight week-old AG129 mice were administered a lethal dose of DENV2 D2S10 intravenously (i.v.) in a volume of 100 μL. At 24 hours p.i., mice were injected one time intraperitoneally (i.p.) with the indicated amounts of antibody in a volume of 200 μL, and 200 μL of PBS was administered to a group of mice as a negative control. Mice were followed for 10 days and observed for morbidity and mortality twice daily. The number of mice per group is indicated in S1 Table. Each experimental condition was tested independently at least twice. Control (PBS and E60 N297Q) animals are the same in panels A and B. P-values indicated with an * are generated by comparing the survival of mice treated with the experimental IgY with survival of mice treated with PBS and p-values indicated with an § are generated by comparing the survival of mice treated with equivalent quantities of either anti-DENV2 IgY or control IgY.* p = 0.0035; ** p = 0.0019; *** p = 0.003; §§ p = 0.04.

AG129 mice were challenged with a lethal dose of D2S10 and the *in vivo* protective capacity of anti-DENV2 IgY was determined. In Fig 4, the results of six different experiments using a lethal dose (1.0x10^7^ PFU) of DENV2 D2S10 were combined. All mice challenged with D2S10 and therapeutically administered PBS as a negative control succumbed to disease by five days post-infection, while all animals infected but treated with positive control mAb E60 N297Q [12] survived (p = 0.0003 as compared to PBS) (Fig 4A and 4B). The E60 N297Q control mAb is different from the E60 mAb used in the *in vitro* studies in that it has been genetically modified to abolish FcγR and C1q binding. Similar to the positive control E60 N297Q mAb, 100% therapeutic efficacy was also observed with 1 mg anti-DENV2 IgY (n = 4) administered 24 hours post-infection (p = 0.0019), while 1 mg of control IgY yielded 25% protection (n = 4, p = 0.04 comparing equal amounts of anti-DENV2 IgY and control IgY) (Fig 4A). Next, we tested multiple decreasing amounts of anti-DENV2 IgY, including doses of 500 ug and 50 ug. Treatment with 500-μg anti-DENV2 IgY (n = 6) provided 66% protection (p = 0.0035), and treatment with 50 μg anti-DENV2 IgY (n = 10) provided 33% protection. These data, including the therapeutic efficacy of 2 mg anti-DENV2 IgY and control IgY, are provided in S1 Table.

### DENV2 IgY epitopes

To determine the specificity of full length and alternatively spliced anti-DENV2 IgY, linear epitope mapping was completed using the DENV2 envelope (E), pre-membrane (prM), nonstructural 1 (NS1) and nonstructural 3 (NS3) proteins in a microarray. Microarray slides were covalently linked with 15-mer peptides with a 11 amino acid overlap, and 3 amino acid offset, spanning the entire sequence of either the E, prM, NS1 and NS3 proteins. Slides were incubated with either naïve IgY, anti-DENV2 full length IgY or alternatively spliced IgY. Reactivity was compared to negative control peptides (scrambled peptide or AAAAAAAAAAAAAAA peptide) on each slide; the average MFI of the negative controls was used for comparison.

**Fig 5:**
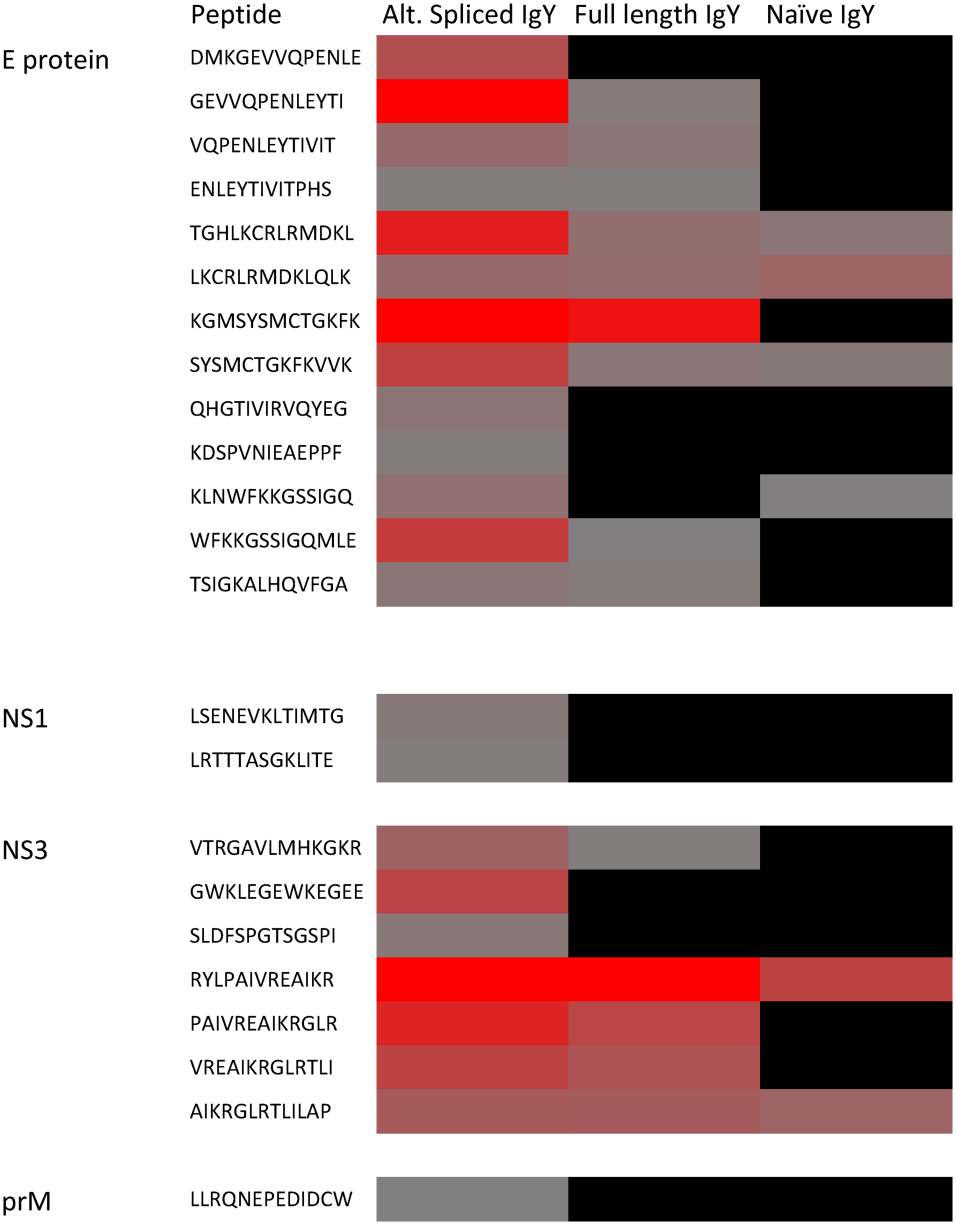
Identification of DENV2 epitopes recognized by IgY. The amino acid sequences of four DENV proteins (E, NS1, NS3, and prM) were used to generate 15-mer overlapping peptides that were spotted onto microarray slides. Slides were incubated with alternatively spliced anti-DENV2 full-length anti-DENV2 IgY, or naïve IgY. Reactivity is measured based on a graded color scale ranging from no reactivity in black to high reactivity (based on highest value) in red. Values represent MFI minus background. Selected peptide amino acid sequences are listed in the order that they would appear in the full-length protein. Not all peptides are included for each protein.

Unbiased heat maps were generated to display the epitope mapping data in order to compare the epitope binding regions between full length, alternatively spliced, and naïve IgY. The MFI is shown in a color gradient, with the red color representing the strongest binding of the IgY antibody to the indicated peptide (Fig 5). Epitopes recognized by full-length anti-DENV2 IgY and alternatively spliced anti-DENV2 IgY were compared to each other and to previously characterized anti-DENV2 IgG epitopes. Our results suggest that the majority of the peptide sequences that were recognized were within the E protein, whereas there were few in the NS1 and NS3 proteins, and one with very low mean MFI in the prM protein. Some of the peptides recognized by the full-length and alternatively spliced anti-DENV2 IgY were similar, while others appeared unique to each of the different antibody populations. Interestingly, some of the peptides recognized by the anti-DENV2 IgY appeared to be distinct from previously described epitopes targeted by either human or mouse anti-DENV2 sera [33–51]. Specifically, we found that the peptide KGMSYSMCTGKFK at position 295–307 in the E protein domain III N-terminal stem region was strongly recognized by both anti-DENV2 full-length and alternatively spliced IgY, whereas alternatively spliced IgY also displayed high reactivity to the peptides GEVVQPENLEYTI at position 127–139 in the E protein domain II and TGHLKCRLRMDKL at position 278–290 in the E protein domain I. Interestingly, both of these regions recognized by alternatively spliced IgY cover hinge regions between the E protein domains.

Naïve IgY recognized some of the same peptides that both full-length and alternatively spliced anti-DENV2 IgY recognized; however, there were more peptides recognized that were unique to both of the DENV2 specific IgY populations. It is also apparent that the anti-DENV2 alternatively spliced IgY recognized more peptides than the full-length anti-DENV2 IgY.

## Discussion

In this study, we demonstrate that anti-DENV2 IgY purified from goose egg yolk is effective in neutralizing DENV2 D2S10 viral infection both *in vitro* and *in vivo* and does not induce ADE. Vaccination with DEVN2 antigen induced a strong humoral immune response in the geese, with antibody titers maintained for over six weeks and reaching as high as 1: 6,400,000. We observed therapeutic efficacy with 1–2 mg anti-DENV2 IgY administered 24 hours post-infection in lethally infected AG129 mice. This protection was comparable to the therapeutic protection observed with the MAb E60-N297Q positive control. Our results also indicate some non-specific protection that may be provided by large amounts of naïve goose IgY, as indicated by both the *in vivo* challenge data and the epitope mapping. Experiments that were performed prior to epitope mapping were completed using unfractionated serum that included both the full-length IgY and alternatively spliced polyclonal antibody populations. Further studies will determine the neutralization capacity of both serum components individually as well as the neutralization capacity of the epitope-specific affinity-purified IgY.

At present, there are no licensed therapies to treat the severe manifestations of DENV infection. It has been suggested that ADE resulting from pre-existing antibodies binding at sub-neutralizing concentrations to heterotypic DENV ultimately results in increased viral load, increased activation of cytokines, and the activation of complement. All of these phenomena increase the likelihood of vascular leakage that may result in mortality if not treated appropriately, and underscore the need to develop therapeutics that will not induce ADE, such as anti-DENV2 IgY. The development of a vaccine has been problematic, in part due to the possible risk of eliciting suboptimal immune responses that will lead to ADE and severe disease following infection with heterologous virulent strains. Passive immunotherapy with neutralizing antibodies may provide an alternative for the treatment of severe dengue. Our data indicate that anti-DENV2 IgY does not induce ADE, presumably because similar to aglycosylated IgG, IgY does not bind FcγRs. This characteristic is especially advantageous because it does not require any genetic modification or engineering to prevent enhancement, unlike other non-avian derived antibody therapeutics [12].

Humanized anti-DENV MAbs obtained from mice or non-human primates have been produced to treat dengue, but the majority of these antibodies are weakly neutralizing and serotype cross-reactive [34, 52, 53]. Potently neutralizing human MAbs are rare, indicating that only a small fraction of the total antibody response during natural infection is responsible for virus neutralization. Here we demonstrate that anti-DENV IgY is able to neutralize DENV infection *in vitro* and *in vivo*. We recognize that the amount of anti-DENV IgY required to induce full protection in the mouse model is much greater than the amount of the E60 N297Q monoclonal antibody. Further characterization of the IgY antibodies to determine which antibodies are responsible for providing protection and generating a more potent anti-DENV IgY cocktail may overcome this limitation. We also recognize that high doses of control IgY induced moderate protection, suggesting that there are nonspecific mechanisms of protection provided by IgY antibodies. One potential explanation may be that administration of large doses of IgY induces an immune response or hypersensitivity reaction in mice that may be facilitating the protection provided by the antibody therapeutic itself. It is unlikely that in the absence of binding to human Fc receptors and complement that either the anti-DENV2 IgY or the naïve IgY promotes serum sickness. It is also possible that there are mammalian receptors that are homologous to avian receptors that could facilitate the binding and activation of the immune system. Furthermore, we also hypothesize that there may be cross-reactivity between DENV2-specific neutralizing epitopes and other viruses or bacteria that these geese may have come in contact with.

Epitope mapping of four complete DENV proteins—E, prM, NS1, and NS3 –suggested that naïve, anti-DENV2 full-length, and anti-DENV2 alternately spliced IgY bind to similar epitopes, but that both anti-DENV2 IgY populations recognize epitopes not recognized by naïve IgY and that they are different from one another. The majority of the epitopes detected were located within the E protein, with some located in the NS3 protein, two located in the NS1 protein, and one with very low mean MFI in the prM protein. These data are consistent with the current literature suggesting that most neutralizing epitopes are located within the E protein. The E protein is located on the surface of the virus and plays a key role in host cell entry. In contrast, the NS3-specific epitopes presented are uncharacteristic. NS3 is a viral protease and helicase that is in part responsible for virus processing and replication, and normally would not induce NS3-specific antibodies during immunization with an inactivated virus, as was used in our immunization protocol. It is also unlikely that these anti-NS3 antibodies alone are able to neutralize DENV [54–56]. The NS1 epitopes were only recognized by the alternatively spliced anti-DENV2 IgY, demonstrating that full-length and alternatively spliced anti-DENV2 IgY bind different epitopes. It is also interesting that the alternatively spliced anti-DENV2 IgY recognized more peptides overall as compared to the full-length anti-DENV2 IgY, and this could be due to both the smaller size of the antibody and the recognition of diverse epitopes.

The reactivity of naïve IgY to DENV2 peptides is surprising but offers insight to the protection provided by naïve IgY in the *in vivo* studies. It is possible that naïve IgY is binding to certain DENV2 peptides due to the nature of the amino acid characteristics, specifically if there are cross-reactive epitopes to other viruses or bacteria. At present, there are no identified mammalian receptors that bind to IgY, but it is also possible that these DENV2 epitopes share biochemical characteristics to a putative IgY receptor.

In mapping linear epitopes, we also recognize that we have potentially missed biologically relevant conformational and structural epitopes that would have been recognized using an alternate epitope mapping approach with intact virions. Recent reports suggest that many DENV specific epitopes rely on a mature viral surface and conformational motions of the virus particle, which are dynamics that are absent in mapping linear epitopes [57]. We also recognize that by using polyclonal antibodies for epitope mapping that we were not able to determine the specific epitopes that are important for virus neutralization and protection. However, because these epitopes have not been previously demonstrated as neutralizing for mammalian or murine MAbs, when comparing other linear neutralizing epitopes [33–51], it is important to consider further characterization of these and other conformational epitopes. At present, our data are consistent with literature demonstrating that IgY recognizes different epitopes than IgG [16, 17], suggesting that the anti-DENV antibody population generated in geese is different from what might be generated during infection or vaccination in humans or mice.

The exploration for alternative therapies is critical in the absence of fully effective DENV vaccines or antivirals, and immunotherapy is a potential candidate. The therapeutic potential of polyclonal avian-derived IgY has been previously established for the treatment of *Pseudomonas aeruginosa*, which is in an ongoing phase III clinical trial under the auspices of the European Medicines Agencies [58–60], in addition to treatment of *Candida albicans*, [61, 62], and many toxins and venoms [63–65]. Although the animal models used in our study and others demonstrate the therapeutic efficacy of IgY, we recognize that there are potential obstacles that may arise in translating IgY therapeutics for human clinical application. The immunogenicity of IgY has been tested previously [66–68] in both pigs and mice. Vega et al. and Torche et al. have both demonstrated that administration of IgY to pigs via both systemic and local routes induced an anti-IgY antibody response, primarily consisting of the IgG subclass. These data suggest that IgY is antigenic and although the biochemical properties of this antibody molecule do not facilitate considerable binding to mammalian Fc receptors, serum sickness is a theoretical possibility if IgY is administered in large amounts. Whether or not IgY elicits an allergic response in pigs is unknown, however Akita et al. demonstrated that administration of egg yolk containing IgY, purified IgY, and IgY Fab’ to mice failed to induce an IgE response. They further determined that there was very little cross reactivity between egg white protein, which is highly allergenic, and purified IgY. Future studies will need to be conducted to elucidate the antigenicity and allerginicty of IgY in more relevant models. In addition to these studies, we must also consider the timing and dose of administration and the myriad of co-morbidities that are present in dengue endemic countries where protection from disease is most needed.

Overall, we determined the therapeutic potential of anti-DENV2 IgY and demonstrated that IgY does not induce ADE. Our results indicate that anti-DENV2 IgY is capable of neutralizing but not enhancing DENV2 infection and that anti-DENV2 IgY therapeutically administered to mice can protect against lethal DENV2 challenge. Further, we have confirmed that the antibody repertoire generated against DENV in geese is different from the repertoire generated by mammals.

## Supporting information

S1 Table In vivo therapeutic efficacy of anti-DV2 IgY. Six-to-eight week-old AG129 mice were administered a lethal dose of DENV2 D2S10 intravenously (i.v.) in a volume of 100 μL. At 24 hours p.i., mice were injected one time intraperitoneally (i.p.) with the indicated amounts of antibody in a volume of 200 μL, and 200 μL of PBS was administered to a group of mice as a negative control. Mice were followed for 10 days and observed for morbidity and mortality twice daily. Percent survival and p values are indicated for each treatment group.
(PDF)
