# Supplementary material for: Dengue virus specific IgY provides protection following lethal dengue virus challenge and is neutralizing in the absence of inducing antibody dependent enhancement
Source: PLoS Negl Trop Dis. 2017 Jul 7;11(7):e0005721. doi: 10.1371/journal.pntd.0005721 (PMC5517069; doi:10.1371/journal.pntd.0005721)
Supplement: S1 Table — Six-to-eight week-old AG129 mice were administered a lethal dose of DENV2 D2S10 intravenously (i.v.) in a volume of 100 μL. At 24 hours p.i., mice were injected one time intraperitoneally (i.p.) with the indicated amounts of antibody in a volume of 200 μL, and 200 μL of PBS was administered to a group of mice as a negative control. Mice were followed for 10 days and observed for morbidity and mortality twice daily. Percent survival and p values are indicated for each treatment group. (PDF) [file pntd.0005721.s001.pdf]

S1 Table: In vivo therapeutic efficacy of anti-DV2 IgY

Experimental Group	Total Number of mice/ group	Number of mice that survived	Survival (%)
2 mg anti-DV2 IgY	12	12	100%
1 mg anti-DV2 IgY	4	4	100%
500 ug anti-DV2 IgY	6	4	67%
50 ug anti-DV2 IgY	10	2	20%
2 mg control IgY	14	8	57%
1 mg control IgY	4	1	25%
PBS	8	0	0%
20 ug E60 N297Q	6	6	100%

Statistical Significance

Experimental Group	PBS	2 mg cntrl IgY	1 mg cntrl IgY
2 mg anti-DV2 IgY	<0.0001	0.0122	
1 mg anti-DV2 IgY	0.0019		0.04
500 ug anti-DV2 IgY	0.0035		
50 ug anti-DV2 IgY	0.2263		
2 mg control IgY	0.0013		
20 ug E60 N297Q	0.0003		
